# mRNA Vaccines against Flaviviruses

**DOI:** 10.3390/vaccines9020148

**Published:** 2021-02-12

**Authors:** Clayton J. Wollner, Justin M. Richner

**Affiliations:** Department of Microbiology and Immunology, University of Illinois College of Medicine, Chicago, IL 60612, USA; cwolln2@uic.edu

**Keywords:** flavivirus, mRNA vaccines, Dengue, Zika, tick-borne encephalitis

## Abstract

Numerous vaccines have now been developed using the mRNA platform. In this approach, mRNA coding for a viral antigen is in vitro synthesized and injected into the host leading to exogenous protein expression and robust immune responses. Vaccines can be rapidly developed utilizing the mRNA platform in the face of emerging pandemics. Additionally, the mRNA coding region can be easily manipulated to test novel hypotheses in order to combat viral infections which have remained refractory to traditional vaccine approaches. Flaviviruses are a diverse family of viruses that cause widespread disease and have pandemic potential. In this review, we discuss the mRNA vaccines which have been developed against diverse flaviviruses.

## 1. Introduction

Flaviviridae is a diverse family of positive sense, RNA viruses that are spread predominantly by arthropod vectors [1]. Outbreaks of flaviviruses across the globe have plagued humankind for centuries [2]. Even in modern times, flaviviral outbreaks can lead to global pandemics as demonstrated after the introduction and subsequent spread of West Nile virus into North America in 1999 and more recently, the emergence of Zika virus into the Western Hemisphere in 2013 [3]. One of the most successful early vaccination campaigns ever was against the flavivirus yellow fever virus in the 1930’s [4]. Since this time, numerous vaccines have been developed against other flaviviruses including Japanese encephalitis virus (JEV), tick-borne encephalitis virus (TBEV), West Nile virus (WNV), Dengue virus (DENV), and Zika virus (ZIKV) [5]. Traditional flaviviral vaccine development has utilized live-attenuated viruses or chemically inactivated viruses [6,7,8]. Recent advances have led to the development of next-generational nucleic acid-based vaccines in which DNA or RNA encoding for viral antigens can be incorporated into a delivery vector and injected into the host. In this review, we will highlight the recent development of mRNA vaccines against flaviviruses. Reviews which highlight and discuss existing vaccines against specific flaviviruses can be found here; YFV [4], JEV [9], TBEV [10], WNV [11], DENV [12,13], and ZIKV [14]. Reviews which discuss considerations for commercialization and large-scale production of mRNA vaccines can be found here [15,16].

## 2. RNA Vaccine Overview

Advances within the last 15 years have led to a rapid improvement of in vitro-synthesized mRNA stability and delivery techniques, leading to a revolution in the vaccine field. mRNA encoding for a viral gene(s) can be in vitro synthesized and administered into a host organism to drive transient expression of the viral protein. The host then mounts an immune response against the exogenous viral protein and establishes protective immunological memory [17]. Often the mRNA is encapsulated within a lipid nanoparticle (LNP) which protects the mRNA from degradation by endogenous host nucleases. Effective mRNA-LNP vaccines have been generated against many diverse viruses in recent years, including influenza virus [18,19,20,21], HIV [22], rabies virus [23,24], chikungunya virus [25] and human cytomegalovirus [26]. Most recently, the first vaccines to be approved for widescale distribution against the novel SARS-CoV-2 outbreak were mRNA-LNP vaccines encoding for the viral spike protein [27,28,29]. These vaccines highlight the advantages of the mRNA-LNP platform for rapid vaccine development against emerging pathogens and the ability to modulate the immune response by manipulating the targeted antigen.

The mRNA-LNP platform has several advantages over other vaccination strategies; it can be repeatedly administered without inducing immunologic memory to the delivery vector, has no chance of incorporating into potential oncogenic sites within the genome, is relatively inexpensive to synthesize large quantities compared to recombinant protein production, and can be easily scaled from production in small laboratories to manufacturing facilities for vaccination campaigns [15,16]. The mRNA included in the vaccine formulation is in vitro synthesized to mimic the host mRNA in order to increase mRNA stability and translation efficiency. Features include the 5′ cap-1 structure, 5′ and 3′ untranslated elements flanking the open reading frame, and a poly(A)_n_ tail at the 3′ end [30,31,32]. These elements ensure protection from endogenous host exonucleases and efficient translation of the antigen. To ensure that encoded proteins are secreted from the host cell, a short signal peptide is cloned at the N-terminus of the coding region. The signal peptide traffics the polyprotein through the secretory pathway for post-translational processing. Injection of naked, unmodified mRNA into a host is rapidly degraded by extracellular RNases resulting in low expression of the gene-of-interest [33]. Previous research has demonstrated that modification of the mRNA through incorporation of naturally occurring nucleoside analogs (such as psuedouridine [Ψ]) [34], purification of the in vitro transcribed mRNA [35,36,37], and encapsulation in a lipid nanoparticle can greatly enhance the cellular uptake of the mRNA leading to prolonged and enhanced protein expression. These mRNA modifications increase transcript stability and limit detection by the innate immune sensing mechanisms which can inhibit protein translation [36,38,39,40,41]. Local or systemic inoculation of the mRNA-LNP results in high levels of exogenous protein expression. Antigen expression can persist up to 10 days post intramuscular or intradermal administration of modified mRNA-LNP vaccines [42]. Though mRNA vaccines incur many advantages, it is difficult to directly compare results seen with mRNA vaccines to other vaccine platforms such as subunit or VLP vaccines. While other vaccine platforms administer a known amount of antigen with each injection, the mRNA delivered as an mRNA vaccine can persists for days following administration and produces an indiscriminate amount of antigen during that time. In most cases, mRNA vaccines do not contain an adjuvant. Instead, the LNP itself can induce an innate immune response, which serves as a self-adjuvant [43,44]. Further research is needed to define the precise mechanism of LNP immune stimulation, as well as the potential advantages of additional adjuvants in vaccine formulation.

Vaccines can also be developed utilizing self-amplifying mRNA (SA-RNA). In this approach the in vitro synthesized mRNA encodes for components of an RNA-dependent RNA polymerase (RDRP), in addition to the viral antigen. The RDRP amplifies the exogenous RNA species inside of the host cell and produces high levels of a sub-genomic RNA encoding for the viral antigen. The SA-RNA can reach higher abundance inside of the host cell and persists for longer periods of time. Luciferase expression was shown to persists for greater than 21 days in mice following SA-RNA intradermal electroporation compared to only 10 days with an mRNA construct coding for the luciferase gene [45]. The structured 5′ and 3′ termini and the double-stranded RNA replication intermediates of the SA-RNA vaccines can be recognized by innate immunity pattern recognition receptors and lead to a type I IFN response. This immune stimulation can serve as a self-adjuvant thereby increasing vaccine immunogenicity. Alternatively, IFN can activate protein kinase R (PKR) which phosphorylates eIF2α and will inhibit protein translation thereby lowering antigen expression [41,46]. Indeed, Zhong et al. found that efficacy against a SA-RNA Zika virus vaccine was greatly diminished in mice with an intact type I IFN response compared to mice lacking the type I IFN receptor (C57BL/6 versus IFNAR1^-/-^) [47]. Research has demonstrated that lowering these innate immune pathways can enhance antigen expression from SA-RNA constructs [48,49,50].

## 3. Flavivirus Molecular Biology

Flaviviruses are comprised of a positive-sense, single-stranded RNA genome of 10–13 kilobases that encodes for a single open-reading frame. Upon translation, the polyprotein is cleaved into ten individual proteins including three structural proteins [premembrane (prM), envelope (ENV), and capsid (CAP)]. Additionally seven nonstructural proteins (NS) are encoded with varying roles within the viral life-cycle; NS1, NS2A, NS2B, NS3, NS4A, NS4B, and NS5 [1,51]. PrM and ENV colocalize within the ER membrane where they interact to form ENV dimers which coalesce with prM to form immature viral particles. Expression of prM and ENV alone is sufficient to form a viral-like particle. Viral-like particles are smaller than infectious viral particles but encompass many of the same tertiary and quaternary epitopes as an infectious viral particle [52]. The particle undergoes further maturation while trafficking through the trans-golgi network (TGN) before secretion from the cell [1]. The immature viral particle has a rough surface which consists of ENV dimers protruding from the viral particle in a herring-bone confirmation and prM protruding from the surface [53]. The pr portion of the prM protein acts as a chaperone during viral processing to prevent the premature fusion of the ENV protein to the host cell membrane in the acidic environment of the TGN [1,54]. PrM is eventually cleaved by the host protease furin and the viral particle egresses as a mature virion with a smooth surface [1,54,55]. Furin-dependent cleavage of prM is often not complete and partially mature particles with both smooth and rough portions of the viral surface can be found readily by electron microscopy of viral preps [56,57]. To enter the cell, cognate viral epitopes interact with host cell receptors resulting in receptor mediated endocytosis and uptake of the flavivirus. The acidic environment of the endosome results in irreversible conformational changes to ENV [58,59]. These conformational changes expose the 98–111 amino acid region of the ENV protein which is referred to as the fusion loop. Partially mature viral particles with prM intact can undergo further maturation and pr cleavage in the acidified endosome leading to complete maturation and exposure of the fusion loop. Once the fusion loop is exposed, it fuses to the endosomal membrane which results in release of the viral genome into the host cell cytoplasm [53].

## 4. Antibody-Dependent Enhancement

Development of vaccines against flaviviruses is complicated by the potential for antibody-dependent enhancement (ADE). ADE is a phenomenon in which sub-neutralizing concentrations of antiviral IgG antibodies increases the percentage of host cells that are productively infected and leads to increased viral load and pathogenesis [53,60,61]. Antibodies bound to the surface of the viral particles are recognized by Fcγ-receptors (FcγR) on myeloid cells. These antigen-presenting cells take up immune-complexed virus via FcγR-mediated endocytosis [54,61,62]. Mutation of the Fc portion of a monoclonal antibody ablates ADE activity [62,63]. In a normal immune response, antigen-presenting cells opsonize antigen in order to process into peptides which can be presented on MHCII molecules at the cell’s surface for subsequent adaptive immune responses. In the case of flaviviruses, however, this FcγR-mediated endocytosis incurs an advantage for the virus. Virions that are bound to antibodies will be more efficiently endocytosed by FcγR-positive cells than unbound virions. In the event that the antibodies neutralize the virus, the virus is destroyed in the acidified endosome. If the antibodies fail to neutralize the virus, the virus will instead escape the endosome, thus leading to an enhanced viral infection and replication in FcγR-expressing cells [53]. Just like a natural infection, the low pH of the late endosome induces the fusion loop to fuse with the endosomal membrane and the viral genome is released into the cytoplasm. In addition to increased viral uptake, FcγR-mediated endocytosis of DENV resulted in diminished antiviral immune responses when compared to receptor-mediated endocytosis which represents another possible contribution to more severe disease [64].

ADE is driven predominantly by antibodies binding epitopes on a partially immature viral particle, most notably the pr epitope of prM and the fusion loop (FL) epitope of ENV [65]. These epitopes are highly conserved across flaviviruses but the antibodies targeting them are poorly neutralizing. Fully immature virions containing no cleaved prM are considered non-infectious as the viral epitopes responsible for viral uptake are not exposed. However, once trafficked into the endosome via FcγR-mediated endocytosis, host furin can complete cleavage turning an immature, less infectious virion into a fully mature, infectious one. Once the acidic endosome exposes the fusion loop, the viral particle can then fuse with the host endosomal membrane and infect the host cell.

A primary ZIKV or DENV exposure can lead to increased risk for severe DENV clinical outcome because of ADE [61,66]. Similarly, in naïve individuals a vaccine can mimic a primary DENV exposure thus sensitizing an individual to a severe DENV infection. Dengvaxia (CYD-TDV) is a live-attenuated vaccine targeting DENV and evaluated in phase III clinical trials. This vaccine, and other live-attenuated vaccines currently in phase III clinical trials, include the native viral prM and ENV sequences which encode for the cross-reactive, poorly neutralizing epitopes implicated in ADE. Indeed, CYD-TDV (Dengvaxia) induced serotype cross-reactive antibodies instead of type-specific neutralizing antibodies [67]. Despite these potential warnings, Dengvaxia was recommended by the WHO for large scale vaccination programs. After release into large populations, epidemiology studies revealed that vaccination increased the hospitalization rate in naïve children upon DENV infection presumably due to cross reactive, sub-neutralizing humoral immune responses and ADE [12,68,69]. The WHO has since revised guidelines and no longer recommends Dengvaxia in seronegative individuals and in no individual below the age of 9 [69]. These results demonstrate that ADE must be evaluated in the context of developing a safe and efficacious flavivirus vaccine.

## 5. Flavivirus mRNA Vaccines

**Figure 1:**
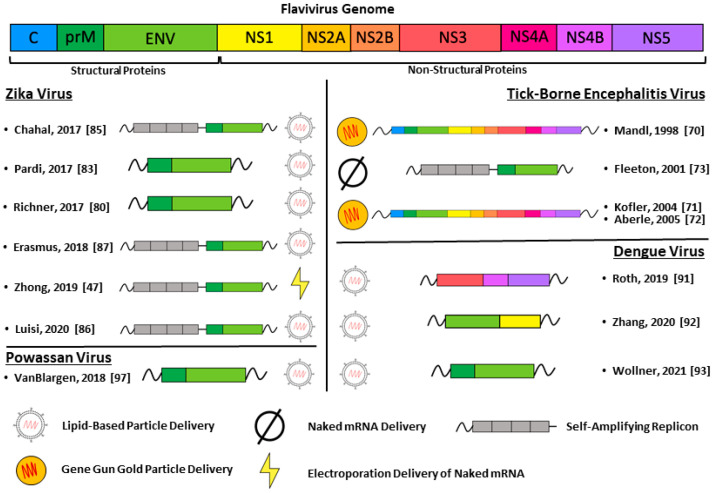
All the different mRNA vaccination strategies discussed in this review are graphically represented. The portion of the viral genome encoded by the mRNA vaccine is matched to the color of the individual viral proteins in the genome. The delivery method for each mRNA vaccine is shown at the bottom of the figure.

mRNA vaccines have been developed against multiple flaviviruses using diverse approaches and varied antigen targets. Vaccines against TBEV, DENV, ZIKV, and Powassan virus are discussed in detail below and highlighted in Figure 1.

### 5.1. Tick-Borne Encephalitis Virus

As early as the 1990s, researchers acknowledged the promise of RNA vaccines. In one of the first RNA vaccine studies, Mandl et al. compared immune response from injection of RNA coding for virulent TBEV to that of RNA coding for an attenuated strain of TBEV [70]. As RNA delivery technology was at its beginning stages, this RNA was delivered via GeneGun of gold particles coated with the RNA. After GeneGun administration of either the virulent RNA or attenuated RNA, antibody response was measured via TBE-antibody ELISA. Antibody response was reported as an ID_50_, or the dose of RNA required to seroconvert 50% of the mice in each group. While vaccination with the virulent strain RNA resulted in an ID_50_ of 0.1 ng, vaccination with the attenuated RNA resulted in a similarly small 0.6 ng [70].

A 2004 study utilizing the same gold particle delivery system characterized the protein and particle expression of a replicating RNA vaccine coding for a whole, live-attenuated TBEV strain [71]. Mutations were introduced into the capsid protein that rendered any sub-viral particles non-infectious. Prime-boost vaccination of BALB/c mice resulted in protection from a lethal challenge and serum neutralizing FRNT50 titers similar to that of mice immunized with whole, inactivated virus (1/20 and 1/80, respectively). Additionally, the IgG titers for RNA immunized mice were similar to mice immunized with whole inactivated virus (1/10,000 in RNA vaccinated mice versus 1/30,000 for inactivated virus) [71]. Administration of 1 μg doses of RNA via prime-boost vaccination produced a greater CD8^+^ T cell response than live virus in a 2005 study by the same group [72]. Additionally, 8 weeks post vaccination, the RNA vaccine resulted in a greater antiviral-IgG titer than live virus with similar, intact IgG titers between the two groups one year after immunization. While all vaccine groups were protected from a lethal viral challenge (100% survival), the RNA vaccinated mice experienced a higher ratio of antiviral IgG2a to IgG1, indicative of a more robust Th1 response [72].

Researchers have even had success administering naked mRNA via intramuscular injection. Louping-ill virus (LIV) is a tick-borne flavivirus infecting sheep closely related to TBEV. A 2001 study by Fleeton et al. incorporated the prM/ENV RNA sequence from LIV into a Semliki Forest virus replicon and administered intramuscularly at a dose of 10 μg in a prime-boost strategy [73]. Vaccination with this mRNA resulted in elevated levels of antigen-specific IgG titer as compared to mice receiving a control replicon coding for LacZ or PBS. The vaccine also conferred protection in an efficacy study with 30% of vaccinated mice succumbing to a lethal challenge of LIV as compared to 100% lethality in the negative control group [73].

### 5.2. Zika Virus

Zika virus is a neurotropic virus first discovered in 1947 in the Zika Forest of Uganda. ZIKV was relatively unstudied until entering the Western Hemisphere in 2013 leading to a global pandemic [3,74]. Spread predominantly by *Aedes aegypti* mosquitos, ZIKV can cause disease in adults ranging from a mild, febrile illness to more severe neurological disorders such as Guillain-Barré syndrome [75,76]. In pregnant women, ZIKV can be vertically transmitted to the fetus leading to developmental defects, spontaneous abortion, and congenital abnormalities such as microcephaly [77,78]. In the face of an ongoing pandemic, the research community quickly developed and tested numerous vaccines across a wide range of platforms against ZIKV, including mRNA vaccines [79]. Here we summarize the findings of six different groups which have independently characterized mRNA vaccines against ZIKV.

In collaboration with Moderna, Richner et al. characterized a 1-methyl pseudouridine mRNA vaccine coding for prM and ENV proteins with the mRNA encapsulated in a lipid nanoparticle. This vaccine elicited a robust immune response in wild-type and immunocompromised mice using a prime-boost strategy with doses as low as 2 μg [80]. Neutralizing EC50 titers of serum 28 days after boost for both a 10 μg and 2 μg dose were as high as 1/100,000. Replacing the IgE signal peptide with a signal peptide derived from JEV led to a 10-fold increase in neutralization antibody titers. The vaccine completely protected both wild-type and immunocompetent mice from a lethal challenge with 100% survival, reduced weight loss, and undetectable viremia as far as 18 weeks post vaccination. Further, infection of the vaccinated mice did not enhance antiviral antibody titers, thus demonstrating sterilizing immunity. In a follow-up study, the ZIKV vaccine was administered into female mice. Mice were then bred to male mice and then challenged with a mouse-adapted ZIKV to model a congenital ZIKV infection. The ZIKV prM/E mRNA vaccine blocked viral vertical transmission and protected against Zika-induced congenital disease with reduced viral titers in the placenta and fetal head. Additionally, fetuses were protected from resorption in the vaccinated mothers [81]. A similar vaccine, mRNA-1893, induced over 90% seroconversion in a phase I/II human clinical trial after a prime-boost vaccination with 10 μg or 30 μg doses [82].

Pardi et al. employed a similar strategy of a nucleoside modified, prM/ENV coding vaccine encapsulated in a LNP, although the vaccine was administered in a single dose [83]. Both C57BL/6 and BALB/c mice that were administered a single, 30 μg dose spread out over four injection sites developed serum neutralizing antibody titers that reached an EC50 of ~1/1000. Furthermore, the C57BL/6 vaccinated mice developed antiviral-specific CD4^+^ T cells. Rhesus macaques were administered a single dose spread out over 10 injection sites. Vaccinated NHPs had a neutralizing antibody response with a PRNT50 of ~1/200 across doses of 50, 200, and 600 μg as well as ZIKV ENV-specific IgG. After vaccination, these NHPs were challenged with ZIKV (PRVABC59) 5 weeks later. Four out of the 5 vaccinated macaques had no detectable viremia over seven days of monitoring [83]. A subsequent study demonstrated that this mRNA vaccine elicited robust germinal center responses characterized by high levels of T follicular helper cells and germinal center B cells [84].

Four separate groups have characterized self-amplifying RNA vaccines against ZIKV. Using a modified dendrimer nanoparticle (MDNP) delivery system to deliver an SA-RNA coding for ZIKV prM/E, Chahal et al. vaccinated mice with 40 μg doses in a prime-boost strategy. Vaccination induced CD8^+^ T cell activation and the authors identified individual T cell epitopes using an envelope peptide library [85].

Zhong et al. developed a SA-RNA vaccine encoding for ZIKV prM/ENV and four non-structural proteins from Venezuelan equine encephalitis virus comprising the RDRP. After administration of naked mRNA via intradermal electroporation in a prime-boost strategy, 75% of BALB/c mice receiving the mRNA vaccine seroconverted as compared to 100% of the mice vaccinated with formalin-inactivated ZIKV (FI-ZIKV). Antigen-specific CD8^+^ and CD4^+^ T cells were induced to a much greater degree in the mRNA vaccinated mice relative to mice receiving FI-ZIKV. IFNAR^-/-^ mice vaccinated with 1 μg of ZIKV prM/ENV SA-RNA in a prime-boost strategy showed complete protection as compared to 60% mortality in mice receiving luciferase SA-RNA negative control vaccine. Additionally, vaccinated mice exhibited less weight loss and reduced viral load after being challenged with ZIKV (MR-766) [47].

A 2020 study by Luisi et al. characterized SA-RNA vaccines coding for variants of ZIKV prM/ENV or CAP/prM/ENV [86]. The in vitro synthesized SA-RNA was packaged into a cationic nano-emulsion that can be mixed shortly before administering into the host. After in vitro studies revealed protein expression from a number of these constructs, vaccination with the prM/ENV constructs induced a neutralizing antibody response in mice with EC50 titers of 1/10,000 when using a 15 μg dose delivered in a prime-boost strategy. Prime-boost vaccination of NHPs with the SA-RNA encoding for the codon-optimized prM/ENV sequence of ZIKV PF/2013 with a JEV signal peptide (VRC5283 SAM) provided the most robust protection after a viral challenge model and significantly reduced viral titers [86].

A 2018 study by Erasmus et al. utilized an alternative lipid carrier amenable to bedside delivery of a SA-RNA vaccine. SA-RNA encoding for ZIKV prM/ENV and the components of the RDRP was in vitro synthesized. The nanostructured lipid carrier (NLC) mixture used by the researchers can be prepared shortly before administering the vaccine with the SA-RNA and lipid components being stored separately allowing for more versatility and flexibility in the storage and transportation of the vaccine components. A single intramuscular injection utilizing an ultra-low 100 ng dose resulted in an impressive PRNT80 of ~1/640 and anti-viral CD8^+^ T cell responses in C57BL/6 mice. Additionally, efficacy studies of vaccinated mice revealed undetectable viremia at 4 dpi and reduced weight loss over 30 days with all vaccinated mice surviving (30 dpi) as compared to 100% mortality of mock vaccinated mice by 10 dpi [87].

ZIKV and DENV are both spread predominantly by *Aedes aegypti* mosquitos and share 40% homology within the ENV protein. Antibodies can cross-react with the conserved epitopes on the surface of these viruses leading to ADE both in vitro and in vivo [88,89]. Indeed, infection with ZIKV can enhance the prevalence of severe DENV infection in humans [66]. Antibodies elicited from a vaccination can also enhance a natural infection through ADE. Serum from mice vaccinated with a mRNA-LNP vaccine encoding for wild-type ZIKV prM/ENV enhanced DENV pathogenesis and disease in both in vitro and in vivo studies. Richner et al. introduced mutations to the fusion loop of the ZIKV ENV protein (ΔFL), an epitope well-characterized to produce antibodies that contribute to ADE. These mutations almost completely ablated in vitro ADE of DENV2 in the serum from the vaccinated mice [80]. Further, serum from the ΔFL vaccinated mice did not enhance DENV disease severity. These results demonstrate that antibody cross-reactivity and potential for DENV disease enhancement should be considered when developing a ZIKV vaccine.

### 5.3. Dengue Virus

Dengue is the most common vector-borne viral infection with a steadily expanding endemic region. There are approximately 390 million dengue infections per year with 40% of the world’s population at risk [90]. *Aedes* species of mosquitos (and predominantly *Aedes aegypti*) spread DENV. Dengue infection is caused by one of four closely related serotypes of dengue virus (DENV1, 2, 3, 4). Development of an effective vaccine against DENV is challenged by the existence of these four distinct serotypes and the cross-reactive immune responses which can elicit ADE (discussed above). Numerous vaccines have been developed against DENV utilizing multiple platforms (see reviews [12,13]). Three groups have recently described DENV vaccines utilizing an mRNA platform.

In a 2019 study, Roth et al. developed a mRNA-LNP vaccine targeting conserved T cell epitopes in the DENV-1 non-structural proteins. The researchers produced a consensus NS protein that was comprised of the most immunogenic portions of NS3, NS4B, and NS5 observed in humans. Prime-boost vaccination of humanized HLA transgenic mice with 10 μg or 2 μg doses resulted in a robust antiviral CD8^+^ T cell response that significantly lowered viral burden upon challenge with a homologous DENV-1 strain. Additionally, due to the homology between portions of the NS proteins across DENV serotypes, the antiviral T cells resulting from vaccination were partially cross-reactive across all four dengue serotypes [91].

In 2020 Zhang et al. developed DENV serotype 2 mRNA vaccines. This study characterized RNA constructs coding for multiple antigens including NS1, prM/ENV (which would produce virus-like particles), or a soluble subunit of ENV protein containing the N-terminal 80% of the ENV protein (E80). Envelope protein was expressed poorly from the prM/ENV construct which yielded poor immunogenicity. Vaccination of BALB/c mice with the mRNA encoding for the soluble portion of DENV-2 ENV (E80) elicited humoral and cell mediated immune responses that protected against a lethal challenge with a homologous serotype of DENV2. Additionally, adding NS1 RNA to the E80 RNA vaccine increased antiviral T cell responses. The DENV-2 E80 mRNA vaccine induced serotype cross-reactive immune responses which resulted in high levels of heterologous ADE of DENV 1, 3, and 4 in an in vitro infection of K562 cells [92].

Recently, our group characterized a DENV serotype 1 mRNA vaccine coding for prM/ENV proteins with promising results [93]. Vaccination induced similar neutralizing antibody responses across high (10 μg) and and low (3 μg) doses delivered via LNP encapsulation and intramuscular injection in a prime-boost strategy. The serum neutralizing EC50 titers reached 1/400, comparable to mice immunized with infectious DENV1 with an EC50 of 1/700. Vaccination also induced anti-DENV1 CD4^+^ and CD8^+^ T cells. Additionally, vaccination with both a wild-type and mutant construct (with the fusion loop epitope eliminated) significantly reduced heterologous ADE of DENV2 in K562 cells. Serum from mice infected with DENV1 infectious virus had 8-fold higher levels of ADE than mRNA vaccinated mice. Vaccination of immunocompromised AG129 mice led to even higher neutralizing antibody titers compared to vaccination of wild-type C57Bl/6 mice (EC50 values of 1/3000 vs. 1/400) presumably due to the lower immunogenic effects of the mRNA itself in the absence of an intact type I IFN response. Efficacy studies utilizing the immunocompromised AG129 mouse model showed the vaccine to be protective both when the AG129 mice were directly vaccinated, and when serum from vaccinated C57BL/6 mice was passively transferred to the AG129 mice [93].

### 5.4. Powassan Virus

Powassan virus (POWV) is a tick-borne flavivirus with human infections reported in North America and Russia. Though POWV infections remain relatively rare, they have increased steadily over the last decade with 13 total states in the USA having confirmed infections [94]. Neuroinvasive POWV infections have a 10% mortality rate with 50% of survivors experiencing long-term neurological effects [95,96]. There are currently no treatments or vaccines available. In a study by VanBlargen et al., a prM/ENV mRNA-LNP vaccine was administered to mice in 10 μg doses in a prime-boost strategy [97]. These mice developed a highly neutralizing antibody titer with post-boost EC50 values as high as 1/100,000. The vaccine protected against both the Spooner strain (vaccine strain) as well as a different strain of POWV (LB) as indicated by survival curves, weight loss curves, and reduced viremia. Serum from vaccinated mice conferred protection in an adoptive transfer study, thereby identifying antibodies as the mechanism of protection. A single, 10 μg dose of the vaccine was sufficient to protect mice. A prime-boost vaccination strategy was also effective against the closely related Langat virus shown by reduced weight loss, reduced clinical signs of infection, and reduced viral load in serum, spleen, and brain after a challenge with Langat virus compared to mice that received a placebo vaccine [97].

## 6. Future Directions

Numerous flaviviruses have the potential to rapidly spread in human populations including Spondweni virus, Usutu virus, Rocio virus, and Powassan virus [98]. The scientific community should be prepared for these potential flaviviral outbreaks by developing effective vaccination strategies before a pandemic occurs. The rapid development of mRNA vaccines to combat the SARS-CoV-2 epidemic proves that the mRNA platform can be quickly implemented in the face of an emergent virus. SARS-CoV-2 was first identified in the human population in December of 2019. Within three months, mRNA vaccines developed by Pfizer/BioNTech and Moderna were being tested in early phase clinical trials [99]. These vaccines achieved regulatory approval within one year of the onset of the pandemic. Researchers have developed forward-looking strategies and vaccines against the threat of some emerging flaviviruses, such as Powassan virus (discussed above). Future endeavors will focus on developing effective vaccines that can be quickly implemented into human clinical trials in the event of an outbreak.

An ideal vaccine could inhibit multiple flaviviruses by targeting conserved epitopes through humoral- and/or cell-mediated immunity. Targeting conserved structural epitopes to induce cross reactive neutralizing antibody responses is challenging due to the diversity in ENV. Flaviviral antibodies which target the conserved ENV dimer epitope (EDE antibodies) can neutralize multiple flaviviruses [100]. The identification of effective mRNA vaccination strategies to deliver these antigens in the appropriate conformation could lead to pan-flaviviral immunity. As an alternative strategy, mRNA vaccines could be designed to elicit T cell responses against the more conserved non-structural proteins. In a short period of time, numerous flaviviral mRNA vaccines have been developed and evaluated in both small animal models, NHP models, and in early phase human clinical trials. The mRNA vaccine platform will continue to evolve and will be a prominent fixture in the vaccine field for many years to come.

## Data Availability

Not applicable.
